# Bridging the 12-6-4 Model and the Fluctuating Charge Model

**DOI:** 10.3389/fchem.2021.721960

**Published:** 2021-07-22

**Authors:** Pengfei Li

**Affiliations:** Department of Chemistry and Biochemistry, Loyola University Chicago, Chicago, IL, United States

**Keywords:** metal ion, force field, molecular dynamics, 12-6-4, fluctuating charge model, ion-induced dipole interaction, metalloproteins

## Abstract

Metal ions play important roles in various biological systems. Molecular dynamics (MD) using classical force field has become a popular research tool to study biological systems at the atomic level. However, meaningful MD simulations require reliable models and parameters. Previously we showed that the 12-6 Lennard-Jones nonbonded model for ions could not reproduce the experimental hydration free energy (HFE) and ion-oxygen distance (IOD) values simultaneously when ion has a charge of +2 or higher. We discussed that this deficiency arises from the overlook of the ion-induced dipole interaction in the 12-6 model, and this term is proportional to 1/*r* ^4^ based on theory. Hence, we developed the 12-6-4 model and showed it could solve this deficiency in a physically meaningful way. However, our previous research also found that the 12-6-4 model overestimated the coordination numbers (CNs) for some highly charged metal ions. And we attributed this artifact to that the current 12-6-4 scheme lacks a correction for the interactions among the first solvation shell water molecules. In the present study, we considered the ion-included dipole interaction by using the 12-6 model with adjusting the atomic charges of the first solvation shell water molecules. This strategy not only considers the ion-induced dipole interaction between ion and the first solvation shell water molecules but also well accounts for the increased repulsion among these water molecules compared to the bulk water molecules. We showed this strategy could well reproduce the experimental HFE and IOD values for Mg^2+^, Zn^2+^, Al^3+^, Fe^3+^, and In^3+^ and solve the CN overestimation issue of the 12-6-4 model for Fe^3+^ and In^3+^. Moreover, our simulation results showed good agreement with previous *ab initio* MD simulations. In addition, we derived the physical relationship between the *C*_4_ parameter and induced dipole moment, which agreed well with our simulation results. Finally, we discussed the implications of the present work for simulating metalloproteins. Due to the fluctuating charge model uses a similar concept to the 12-6 model with adjusting atomic charges, we believe the present study builds a bridge between the 12-6-4 model and the fluctuating charge model.

## Introduction

Metal ion plays significant roles in various biological processes (Thomson and Gray, 1998; Andreini et al., 2004; Woodson, 2005; Waldron and Robinson, 2009; Kepp, 2012). Molecular dynamics (MD) simulations has become an important tool for studying biomolecules (Duan and Kollman, 1998; Lindorff-Larsen et al., 2011; De Vivo et al., 2016; Hollingsworth and Dror, 2018). It can provide information with atomic details and high time resolution. Meaningful MD simulations require reliable models and parameters, but it is challenging to develop accurate models for metal ions (Li and Merz, 2017). Different force field models have been developed for simulating metal ions in biological systems. For example, the bonded model, (Peters et al., 2010), the cationic dummy atom model, (Duarte et al., 2014), the nonbonded model, (Li et al., 2013), and the polarizable models (Sakharov and Lim, 2009; Zhang et al., 2012). Among these models, the nonbonded model is one of the most widely used models because of its simplicity and transferability.

In the past decade, Li, Merz, and co-workers found that the widely used 12-6 Lennard-Jones nonbonded model could not reproduce the experimental hydration free energy (HFE) and ion-oxygen distance (IOD) simultaneously when metal ion has a charge of +2 or higher (Li et al., 2013; Li and Merz, 2014; Li et al.,2015a; Li et al., 2015b). They attributed this deficiency to that the 12-6 nonbonded model did not consider the ion-induced dipole interaction. Because the ion-induced dipole interaction is proportional to 1/*r* ^4^, where *r* is the atomic distance, they proposed the 12-6-4 model and parameterized it for various atomic ions (Li and Merz, 2014; Li et al.,2015a; Li et al., 2015b). The 12-6-4 model could accurately reproduce the experimental HFE and IOD simultaneously after careful parameterization, which successfully solved the deficiency of the 12-6 model (Li and Merz, 2014; Li et al., 2015a; Li et al., 2015b). Moreover, Merz and co-workers have showed that the 12-6-4 model could well simulate the chelate effect (Sengupta et al., 2018) and the thermodynamics of ion binding in a metalloprotein system (Song et al., 2020). However, it was found that for some highly charged metal ions such as Fe^3+^ and In^3+^, the coordination number (CN) value was overestimated by the 12-6-4 model (Li et al., 2015a). For example, Fe^3+^ has an experimental CN of 6, (Marcus, 1988), but the 12-6-4 model predicted its CN value as 6.8–6.9 when using the TIP3P, SPC/E, or TIP4P_EW_ water model (Li et al., 2015a). Recently, Li, Merz, and co-workers have parameterized the 12-6-4 for various ions in conjunction with four new water models (Li et al., 2020; Li et al., 2021; Sengupta et al., 2021). But this issue still exists: the 12-6-4 model predicted a CN of 6.4–6.7 for Fe^3+^ when using the OPC3, OPC, TIP3P-FB, or TIP4P-FB water model (Li et al., 2021). It was proposed that this artifact was due to an inaccurate description of the water-water interactions inside the first solvation shell, which effect is not significant for monovalent and divalent metal ions but becomes more severe for the highly charged ions (Li et al., 2015a). To be more specific, a metal ion with a higher charge will cause larger induced dipoles of the first solvation water molecules, yielding a stronger repulsion between these water molecules. However, the current 12-6-4 model implementation does not take this effect into account, although it considers the ion-induced dipole interactions between the metal ion and surrounding water molecules.

In the present study, we tried to solve the CN overestimation issue by using a 12-6 model with increasing the dipole moments of the first solvation shell water molecules to account for the ion-induced dipole effect. Specifically, we studied the ion-aqueous systems containing Mg^2+^, Zn^2+^, Al^3+^, Fe^3+^, and In^3+^. We showed that this treatment could well reproduce the experimental HFEs and IODs for these ions, and solve the CN overestimation issue for Fe^3+^ and In^3+^. The adjusted dipole moments of the first solvation shell water molecules for different ions are consistent with theory and *ab initio* molecular dynamics (AIMD) simulations. In addition, we derived the physical relationship between the *C*_4_ parameter and induced dipole moment, which has excellent agreement with the simulation results we got. The concept and strategy of the current study can be further applied to biological systems such as metalloproteins. Considering the present study used a similar scheme to the fluctuating charge model, we believe it can bridge the 12-6-4 model and the fluctuating charge model.

## Computational Methods

The potential function of the 12-6 Lennard-Jones nonbonded model in the AMBER force field (Cornell et al., 1995) is shown below:

$$U_{ij}\left(r_{ij}\right)=\frac{e^{2}Q_{i}Q_{j}}{r_{ij}}+\frac{C_{12}^{ij}}{r_{ij}^{12}}-\frac{C_{6}^{ij}}{r_{ij}^{6}}=\frac{e^{2}Q_{i}Q_{j}}{r_{ij}}+\varepsilon_{ij}\left[{\left(\frac{R_{min,ij}}{r_{ij}}\right)}^{12}-2{\left(\frac{R_{min,ij}}{r_{ij}}\right)}^{6}\right]\;$$ (1)

The potential function of the 12-6-4 nonbonded model (Li and Merz, 2014; Li et al., 2015a; Li et al., 2015b) is shown as follows:

$$U_{ij}\left(r_{ij}\right)=\frac{e^{2}Q_{i}Q_{j}}{r_{ij}}+\frac{C_{12}^{ij}}{r_{ij}^{12}}-\frac{C_{6}^{ij}}{r_{ij}^{6}}-\frac{C_{4}^{ij}}{r_{ij}^{4}}=\frac{e^{2}Q_{i}Q_{j}}{r_{ij}}+\varepsilon_{ij}\left[{\left(\frac{R_{min,ij}}{r_{ij}}\right)}^{12}-2{\left(\frac{R_{min,ij}}{r_{ij}}\right)}^{6}\right]-\frac{C_{4}^{ij}}{r_{ij}^{4}}$$ (2)

In these equations, $U_{ij}$ is the potential energy between atoms *i* and *j*. $r_{ij}$ is the distance between the two atoms. $Q_{i}$ and $Q_{j}$ are the particle charges of atoms *i* and *j*, respectively. *e* is the charge of the proton. $C_{12}^{ij}/r_{ij}^{12}$ is the Pauli repulsive term, $-C_{6}^{ij}/r_{ij}^{6}$ is the dispersion attractive term, while $-C_{4}^{ij}/r_{ij}^{4}$ is the ion-induced dipole term. $R_{min,ij}$ is the distance between atoms *i* and *j* where the Lennard-Jones potential has its minimum, while $\varepsilon_{ij}$ is the well depth of the Lennard-Jones potential at this distance. The Berthelot-Lorentz (LB) combining rules are used in the AMBER force field: (Cornell et al., 1995):

$$R_{min,ij}=\frac{R_{min,ii}}{2}+\frac{R_{min,jj}}{2}$$ (3)

$$\varepsilon_{ij}=\sqrt{\varepsilon_{i}\times \varepsilon_{j}}$$ (4)

In the current study, the OPC3 water model was used to simulate the solvent as it represents an accuracy limit for the rigid three-point water models.(Izadi and Onufriev, 2016). The thermodynamic integration (TI) method (Kollman, 1993) was employed to calculate the Δ*G* values. Calculations of the Δ*G* values were performed under the NVT ensemble by assuming that the Gibbs free energy change is identical to the Helmholtz free energy change. The IOD and CN values were obtained based on the radial distribution function (RDF) between the metal ion and water oxygen atoms. Specific steps are described as follows.

For the 12-6 model with regular charges, we calculated the HFE, IOD, and CN values with the method described previously (Li et al., 2020, Li et al., 2021; Sengupta et al., 2021). Among them the regular MD simulations were used to obtain the IOD and CN values.

For the 12-6 model with updated charges, we calculate the HFE, IOD, and CN values as described below. By treating the last snapshot of the regular MD simulations mentioned above as initial structure, we performed a set of TI calculations to obtain Δ*G*_1,ind_ for increasing the dipole moments of the six closest water molecules from the metal ion. Afterwards, we performed a set of TI calculations in the reverse manner to get Δ*G*_2,ind_ for decreasing the dipole moments of these water molecules to the original value. The final HFE was calculated as the sum of the HFE for the 12-6 model with regular charges and (Δ*G*_1,ind_-Δ*G*_2,ind_)/2. These TI calculations were performed with linear scaling: $U\left(\lambda \right)=\left(1-\lambda \right)U_{0}+\lambda U_{1}$. With $U_{0}$ and $U_{1}$ represent the initial and final Hamiltonians, respectively. Seven λ windows (with λ = 0.02544, 0.12923, 0.29707, 0.5, 0.70292, 0.87076, and 0.97455) were used for each set of TI calculations, with each window had 100 ps equilibration and 200 ps production. After finishing the TI calculations, the Gaussian quadrature was used to calculate Δ*G*_1,ind_ and Δ*G*_2,ind_: $\Delta G=\sum_{i}w_{i}\langle \partial U/\partial \lambda \rangle_{i}$. Herein, $w_{i}$ is the weight for window *i*, while $\langle \partial U/\partial \lambda \rangle_{i}$ is the averaged ∂U/∂λ value for window *i*. Considering the uncertainties of the calculated HFEs are ∼1 kcal/mol for monovalent and divalent metal ions and ∼2 kcal/mol for the highly charged metal ions when using the 12-6 model with regular charges, (Li et al., 2013; Li et al., 2015a; Li et al., 2015b), we estimated the uncertainties of the final HFE values are ∼3 kcal/mol or less based on the uncertainty propagation, by assuming the additional TI calculations provide an uncertainty of similar magnitude.

For Fe^3+^ and In^3+^, a weak restraint was used for the additional TI calculations due to there was a CN switching: the CN value was eight or so when these water molecules have fixed charges of the OPC3 water model, while it changes to six for the equilibrated structure of the system when the six closest water molecules to the ion have enlarged dipole moments. To prevent these six water molecules leaving the first solvation shell (due to they have “induced dipoles” which are caused by their proximity to the ion), when calculating Δ*G*_1,ind_ and Δ*G*_2,ind_, a one-side weak restraint of $U=k{\left(r-r_{eq}\right)}^{2}$ with *k* equals 10 kcal/(mol⋅Å^2^) was applied to each of these six ion-oxygen distances when *r* is bigger than 2.5 Å. The same weak restraint was applied to the initial and final states during the TI calculations. Because the CN switching was involved, we carried out longer simulations with each λ window had 200 ps equilibration and 800 ps production for the additional TI calculations of Fe^3+^ or In^3+^.

We noticed that there were free energy changes by applying the restraints mentioned above. To calculate these free energy changes (that is the free energy change between the systems with and without restraints for each of the system with regular charges and the system with updated charges), the free energy perturbation (Zwanzig, 1954) equation was used (with assuming the ensembles A and B are the same):

$$\Delta G_{\text{A}\to \text{B}}=\langle {\mathrm{G\rangle}}_{\text{B}}-\langle {\mathrm{G\rangle}}_{\text{A}}=k_{\text{B}}T\ln\langle e^{\left(E_{\text{B}}-E_{\text{A}}\right)/k_{\text{B}}T}\rangle_{\text{B}}$$ (5)

Where A represents the system without the restraints, and B represents the system with the restraints. Based on Eq. 5, we obtained the $\Delta G_{\text{A}\to \text{B}}$ value for the system with regular charges (herein termed as $\Delta G_{\text{nores}\to \text{res}}^{\text{regularQ}}$), and the $\Delta G_{\text{A}\to \text{B}}$ value for the system with updated charges (termed as $\Delta G_{\text{nores}\to \text{res}}^{\text{updateQ}}$). For each of the system with regular charges and the system with updated charges, by starting from the same final structure of the regular MD simulations mentioned above, 2 ns NPT equilibration plus 2 ns NVT production were performed, with the one-side restraints (mentioned in the last paragraph) applied to both the equilibration and production. The six ion-oxygen distances were collected every 10 fs, and they were used to calculate the $E_{\text{B}}-E_{\text{A}}$ values afterwards, providing 200,000 data points for calculating each of $\Delta G_{\text{nores}\to \text{res}}^{\text{regularQ}}$ and $\Delta G_{\text{nores}\to \text{res}}^{\text{updateQ}}$. Both these two ΔG values should be non-negative as it is always true that $E_{\text{B}}\geq E_{\text{A}}$. Our calculations showed that the result of $\Delta G_{\text{nores}\to \text{res}}^{\text{regularQ}}-\Delta G_{\text{nores}\to \text{res}}^{\text{updateQ}}$ is below 1 kcal/mol for Fe^3+^ or In^3+^, which is below the uncertainty of the calculated HFEs, so we did not account these two ΔG values in the final reported HFE values.

Moreover, also by starting from the final structure of the regular MD simulations, we increased the dipole moments of the six closest water molecules of the metal ion by adjusting their atomic charges, and then we performed 2 ns NPT equilibration with reassigned initial velocities. Then we carried out 2 ns NVT production simulation with the snapshots saved every 500 fs. No restraints were applied in these MD simulations. Afterwards, the RDF between the metal ion and water oxygen atoms was calculated based on the production trajectory. Finally, the IOD and CN values were calculated based on this RDF as we obtained the IOD and CN values for the system with regular charges.

A quasi-cubic water box with a side length of 40 Å and periodic boundary conditions were used in our MD simulations (including the TI simulations). The particle mesh Ewald (PME) method (Darden et al., 1993; Essmann et al., 1995) was used to deal with the long-range electrostatic interactions. A cut-off of 10 Å was used for the nonbonded interactions in the real space. The size of the charge grid was set to 48 in each of the three dimensions in the reciprocal space. A time-step of 1 fs was used, and the three-point “SHAKE” algorithm (Miyamoto and Kollman, 1992) was employed to constrain the geometries of the water molecules with a tolerance of 10^−5^ Å for each bond. The Langevin algorithm with a collision frequency of 2 ps^−1^ was used to control the temperature. The Brenden’s barostat with a relaxation time of 1 ps was employed to control the pressure in the MD simulations in the NPT ensemble. All the simulations were performed using the pmemd. cuda (Salomon-Ferrer et al., 2013; Lee et al., 2020) program in the AMBER software package (Assisted Model Building with Energy Refinement (AMBER), RRID:SCR_014230, and AmberTools, RRID:SCR_018497) (Case et al., 2020).

## Results and Discussion

### Experimental Data

The experimental HFE, IOD, and CN values for the Mg^2+^, Zn^2+^, Al^3+^, Fe^3+^, and In^3+^ ions are shown in Table 1. The Marcus HFE set (Marcus, 1991) was used is because it could be considered as a set of “intrinsic” HFEs, which only considers the free energy change caused by the ion-water interactions but not accounts the free energy change for ion crossing the gas-liquid interface. This is consistent with the free energy calculations performed in this study that used periodic boundary conditions, as discussed previously (Li et al., 2020; Sengupta et al., 2021).

**TABLE 1:** Experimental HFE, IOD, and CN values for Mg^2+^, Zn^2+^, Al^3+^, Fe^3+^, and In^3+^.

	HFE (kcal/mol)*^a^*	IOD (Å)*^b^*	CN*^b^*
Mg^2+^	−437.4	2.09 ± 0.04	6
Zn^2+^	−467.3	2.09 ± 0.06	6
Al^3+^	−1,081.5	1.88 ± 0.01	6
Fe^3+^	−1,019.4	2.03 ± 0.01	6
In^3+^	−951.2	2.15	6

a From Marcus (1991).

b From Marcus (1988).

### CN Overestimation Issue of the 12-6-4 Model

Previously, we have shown that when in conjunction with the OPC3 water model, the 12-6 model could not simultaneously reproduce the experimental HFE and IOD values for each of Mg^2+^, Zn^2+^, Al^3+^, Fe^3+^, and In^3+^ (Li et al., 2020; Li et al., 2021). Specifically, when the 12-6 model was able to reproduce the experimental HFE, it underestimated the IOD by 0.11, 0.22, 0.52, 0.48, and 0.31 Å for Mg^2+^, Zn^2+^, Al^3+^, Fe^3+^, and In^3+^, respectively (Li et al., 2020; Li et al.,2021). When the 12-6 model was able to reproduce the experimental IOD, it provided the HFE less negative than the experimental value by 33, 63, 152, 177, and 126 kcal/mol for Mg^2+^, Zn^2+^, Al^3+^, Fe^3+^, and In^3+^, respectively (Li et al., 2020; Li et al., 2021). Moreover, we have also parameterized the 12-6-4 model for these ions, which showed significant improvement than the 12-6 model: the 12-6-4 model could simultaneously reproduce the experimental HFE and IOD values for each of these ions (Li et al., 2020; Li et al., 2021). However, the 12-6-4 model overestimated the CN values for some highly charged ions (Li et al., 2015a; Li et al., 2021). In Table 2 we showed the 12-6-4 parameters for Mg^2+^, Zn^2+^, Al^3+^, Fe^3+^, and In^3+^ in conjunction with the OPC3 water model, and the simulated HFE, IOD, and CN values from the previous studies (Li et al., 2020; Li et al., 2021). It can be seen that the 12-6-4 model overestimated the CN values of Fe^3+^ and In^3+^ as 6.7 and 8, which is significantly higher than the experimentally determined CN values of 6 (see Table 1). In addition, to better understand the influence of the *C*_4_ term, we calculated the HFE, IOD, and CN values by ignoring the *C*_4_ term in the potential (i.e. using the regular 12-6 model with the same Lennard-Jones parameters) in the current study. These results are shown in Table 2 as well. We can see that when ignoring the *C*_4_ term, the simulated HFE changed to less negative by 46, 78, 201, 195, and 139 kcal/mol for Mg^2+^, Zn^2+^, Al^3+^, Fe^3+^, and In^3+^, respectively. Meanwhile, the simulated IOD increased by 0.04, 0.06, 0.07, 0.13, and 0.06 Å for Mg^2+^, Zn^2+^, Al^3+^, Fe^3+^, and In^3+^, respectively. These values highlighted the importance of the *C*_4_ term. Moreover, the CN value of the Fe^3+^ ion increased from 6.7 to 8.1, showing an enlarged overestimation when ignoring the *C*_4_ term.

**TABLE 2:** The 12-6 and *C*_4_ parameters and the simulated HFE, IOD, and CN values for Mg^2+^, Zn^2+^, Al^3+^, Fe^3+^, and In^3+^ in conjunction with the OPC3 water model.^a^

	*R*_min_/2 (Å)	*ε* (kcal/mol)	*C*_4_ (kcal/mol⋅Å^4^)	HFE (kcal/mol)	IOD (Å)	CN
Mg^2+^	1.433	0.02174524	117	−438.0	2.10	6.0
Mg^2+^	1.433	0.02174524	N/A	−392.0	2.14	6.0
Zn^2+^	1.441	0.02343735	199	−467.3	2.09	6.0
Zn^2+^	1.441	0.02343735	N/A	−389.8	2.15	6.0
Al^3+^	1.361	0.01031847	363	−1081.3	1.88	6.0
Al^3+^	1.361	0.01031847	N/A	−882.0	1.95	6.0
Fe^3+^	1.455	0.02662782	429	−1019.0	2.03	6.7
Fe^3+^	1.455	0.02662782	N/A	−824.3	2.16	8.1
In^3+^	1.487	0.03507938	330	−951.4	2.16	8.0
In^3+^	1.487	0.03507938	N/A	−812.0	2.22	8.0

a The 12-6-4 parameters and simulated HFE, IOD, and CN values for Mg^2+^ and Zn^2+^ are from Ref. (Li et al., 2020). The 12-6-4 parameters and simulated HFE, IOD, and CN values for Al^3+^, Fe^3+^, and In^3+^ are from Ref. (Li et al., 2021).

### Solution of the CN Overestimation Issue

The CN overestimation issue of the 12-6-4 model not only exists for Fe^3+^ and In^3+^ but also for other highly charged metal ions such as Tl^3+^ (Li et al., 2015a; Li et al., 2021). We have discussed that this artifact was caused by the fixed charge nature of the first solvation water molecules. As the ion charge increases, the first solvation shell water molecules should have larger induced dipole moments caused by the ion-induced dipole interaction, yielding a stronger repulsion between them. However, this behavior has not been considered by the current 12-6-4 scheme, which only takes into account the ion-induced dipole interaction between ion and water molecules but not considers its influence on the water-water interactions. Hence, to solve the CN overestimation issue, we increased the dipole moments of the first solvation shell water molecules explicitly to account for their enlarged dipole moments caused by the ion-induced dipole interaction. We understood that this strategy was not perfect as it did not consider the charge fluctuations along with each subtle configuration change inside the first solvation shell and did not change the water dipole moments in the second solvation shell or beyond. However, we believed that this strategy could well serve for a qualitative and demonstration purpose. For example, the *ab initio* MD simulations indicated that the water molecules in the second solvation shell of Zn^2+^, Al^3+^, and Fe^3+^ had almost identical dipole moments as those of bulk water molecules (Bogatko et al., 2010; Cauët et al., 2010).

By adjusting the dipole moments of the water molecules in the first solvation shell (i.e., the six water molecules which are closest to the metal ion), we were able to reproduce the experimental HFEs within 1 kcal/mol by using the 12-6 model. Using the same water charge parameters, we calculated the IOD and CN values as well. The parameters and simulated results are shown in Table 3. The Lennard-Jones parameters of metal ions employed in these simulations are the same as described in Table 2. We can see that these parameters could well reproduce the experimental IOD and CN values with reasonable accuracy as well. Specifically, in order to reproduce the experimental HFEs, we need to increase the dipole moments of the first solvation shell water molecules by 0.34, 0.58, 0.81, 0.89, and 0.70 D for Mg^2+^, Zn^2+^, Al^3+^, Fe^3+^, and In^3+^ respectively. This is consistent with the trend that ion with a higher charge will cause larger induced dipoles of the surrounding molecules, and Zn^2+^ has a stronger such ability than Mg^2+^ (by considering Zn^2+^ has the same IOD but a more negative HFE than that of Mg^2+^, see Table 1).

**TABLE 3:** Simulated HFE, IOD, and CN values when using the 12-6 model with the adjusted charges for the six closest molecules to the metal ion.

	*R*_min_/2 (Å)	*ε* (kcal/mol)	*Q*_O_ (*e*)*^a^*	*Q*_H_ (*e*)*^a^*	$\mu_{\text{tot}}$ of Water (D)^*a*^	$\mu_{\text{ind}}$ of Water (D)^*a*^	HFE (kcal/mol)	IOD (Å)	CN
Mg^2+^	1.433	0.02174524	−1.02	+0.51	2.77	0.34	−437.5	2.12	6.0
Zn^2+^	1.441	0.02343735	−1.1096	+0.5548	3.01	0.58	−467.5	2.12	6.0
Al^3+^	1.361	0.01031847	−1.194	+0.597	3.24	0.81	−1,082.1	1.92	6.0
Fe^3+^	1.455	0.02662782	−1.222	+0.611	3.32	0.89	−1,019.0	2.02	6.0
In^3+^	1.487	0.03507938	−1.152	+0.576	3.13	0.70	−951.9	2.07	6.0

^a^For the OPC3 water model, the charge of the water oxygen atom is −0.8952 *e*, the charge of the water hydrogen atom is +0.4476 *e*, and the dipole moment of the water molecule is 2.43 D (Izadi and Onufriev, 2016).

### Induced Dipole Moments Predicted by Theory and Quantum Calculations

To better understand the ion-induced dipole interaction, we performed the following calculations. The induced dipole of a particle caused by a point charge can be calculated based on the following equation:

$$\mu_{\text{ind}}=\alpha E=\alpha \frac{q}{4\pi \varepsilon_{0}\varepsilon_{r}r^{2}}=\frac{\alpha_{0}q}{\varepsilon_{r}r^{2}}$$ (6)

Where $\mu_{\text{ind}}$ is the induced dipole, α is the polarizability of the particle, *E* is the electric field strength act on that particle, *q* is the charge magnitude of the point charge, *r* is the distance between the point charge and particle, $\varepsilon_{0}$ is the vacuum permittivity, $\varepsilon_{r}$ is the dielectric constant of the medium, $\alpha_{0}$ is the polarizability volume of the particle which equals $\alpha /4\pi \varepsilon_{0}$. The polarizability volume of a water molecule was determined to be 1.444 Å^3^ experimentally (Eisenberg and Kauzmann, 1969). We can calculate its $\mu_{\text{ind}}$ when there is a monovalent ion placed 2.05 Å away from it and $\varepsilon_{r}=1$ (as used in the AMBER force field (Cornell et al., 1995)) based on Eq. 6:

$$\mu_{\text{ind}}=\frac{\alpha_{0}q}{\varepsilon_{r}r^{2}}=\frac{1.444\times \left(+1e\right)}{{\left(2.05\right)}^{2}}\;Å\cdot e=1.65\;\text{D}$$ (7)

The permanent dipole moment $\mu_{\text{per}}\;$of a water molecule was determined to be 1.85 D under gas phase (Dyke and Muenter, 1973). So this predicts that the total dipole moment $\mu_{\text{tot}}$ (i.e., $\mu_{\text{per}}+\mu_{\text{ind}}$) would be 3.5 D for the water molecule inside a monovalent ion-water complex with the ion-oxygen distance as 2.05 Å, by assuming both $\mu_{\text{ind}}$ and $\mu_{\text{per}}$ point towards the monovalent ion. Because $\mu_{\text{ind}}$ is proportional to q, so we can get the $\mu_{\text{tot}}$ of the water molecule is 5.15 and 6.80 D in the analogous divalent ion-water complex and trivalent ion-water complex, respectively.

The above theoretical calculations assumed the water molecule was a single-point particle. Although it could provide some insights about the magnitude of $\mu_{\text{ind}}$, this description was not accurate. To further evaluate $\mu_{\text{ind}}$ of the water molecule in an ion-water complex, we performed quantum calculations for the dipole moment of a water molecule when placing a point charge on the central axis of the water molecule. The quantum calculations were performed using the B3LYP density functional (Becke, 1988; Lee et al., 1988; Becke, 1993; Stephens et al., 1994) with the 6-31G** basis set (Hehre et al., 1972; Hariharan and Pople, 1973). To keep consistent with the MD study, geometry of the OPC3 water model was used for the water molecule. These quantum chemistry calculations were performed using the Gaussian16 software (Gaussian, RRID:SCR_014897) (Frisch et al., 2019). Herein, we put the point charge 2.05 Å away from the coordinating water oxygen atom. The calculated results are shown in Table 4. We can see that when the point charge has a magnitude of +1 *e*, the induced dipole of the water molecule is 0.78 D (with subtracting 1.96 from 2.74 D). This value is only about half of the value calculated by using Eq. 6. However, in terms of the correlation between $\mu_{\text{ind}}$ and q, we can see a clear trend which is consistent with Eq. 6 that $\mu_{\text{ind}}$ is proportional to q. Moreover, to test the dependence of the calculated values on density functionals, we also calculated these values by using the PBE0 density functional (Perdew et al., 1996; Perdew et al., 1997; Adamo and Barone, 1999) with the 6-31G** basis set. The results are shown in Table 4 as well. We can see these values are very close to what we obtained by using the B3LYP density functional.

**TABLE 4:** $\mu_{\text{tot}}$ and $\mu_{\text{ind}}$ calculated at the B3LYP/6-31G** and PBE0/6-31G** (values before and after slashes, respectively) levels of theory when placing a point charge on the central axis of a water molecule with OPC3 geometry and 2.05 Å away from the coordinating water oxygen.

Point charge (*e*)	$\mu_{\text{tot}}$ (D)	$\mu_{\text{ind}}$ (D)
0	1.96/1.99	0.00/0.00
+1	2.74/2.77	0.78/0.78
+2	3.47/3.49	1.51/1.50
+3	4.17/4.19	2.21/2.20

Although having a quantum description of the water molecule, the above quantum calculations still use a point-charge representation for the metal ion. Furthermore, we did a literature investigation about previous work of AIMD simulations for metal ions in aqueous phase. We found that the magnitudes of the dipole enlargements for the first solvation shell water molecules of Fe^3+^ agreed well with our parameters shown in Table 3. Specifically, previous AIMD simulations of the Zn^2+^-aqueous system indicated the dipole moments of first solvation shell water molecules are 0.7 D larger than those of those of bulk water molecules (Cauët et al., 2010). Other studies indicated that this value is 0.2 D for the Mg^2+^ ion (Lightstone et al., 2001) and 1.0 D for the Al^3+^ ion (Bogatko et al., 2010). All of these values have excellent agreement with our parameters in Table 3. Previous AIMD simulations found that the first solvation shell water molecules of Fe^3+^ had dipoles of 0.5 D higher than those of bulk water molecules, (Bogatko et al., 2010), which only qualitatively agreed with our value in Table 3. This may be due to the challenge of simulating the d-orbital effect by using the classical force field. In general, the strategy employed in the current study not only reproduced multiple physical properties simultaneously through a physically meaningful way but also showed well agreement with AIMD simulations.

### Relationship Between the *C*_4_ and $\mu_{ind}$ Values

To elucidate the relationship between the *C*_4_ parameter and $\mu_{\text{ind}}$, we performed the following derivation. The ion-induced dipole interaction potential can be described by the following equation:

$$U\left(r\right)=-\overset{E}{\underset{0}{\int }}\mu_{\text{ind}}dE=-\overset{E}{\underset{0}{\int }}\alpha EdE=-\alpha \overset{E}{\underset{0}{\int }}EdE=-\frac{1}{2}\alpha E^{2}=-\frac{1}{2}\alpha {\left(\frac{q}{4\pi \varepsilon_{0}\varepsilon_{r}r^{2}}\right)}^{2}=-\frac{1}{2}\frac{\alpha_{0}q^{2}}{4\pi \varepsilon_{0}\varepsilon_{r}^{2}r^{4}}$$ (8)

Herein, U(r) represents the energy needed to bring a neutral species from infinite away where the electric field strength equals zero to a certain position with an electric field strength of *E*. Because the ion-induced dipole term equals $-C_{4}/r^{4}$ in the 12-6-4 model, we can get:

$$C_{4}=\frac{1}{2}\frac{\alpha_{0}q^{2}}{4\pi \varepsilon_{0}\varepsilon_{r}^{2}}$$ (9)

Moreover, we can obtain the following relationship based on Eq. 6:

$$\mu_{\text{ind}}\varepsilon_{r}r^{2}=\alpha_{0}q$$ (10)

By combining Eqs 9 and 10, we can obtain:

$$C_{4}=\frac{1}{2}\frac{\alpha_{0}q^{2}}{4\pi \varepsilon_{0}\varepsilon_{r}^{2}}=\frac{1}{2}\frac{\mu_{\text{ind}}r^{2}q}{4\pi \varepsilon_{0}\varepsilon_{r}}$$ (11)

We denote:

$$k_{e}=\frac{1}{4\pi \varepsilon_{0}\varepsilon_{r}}$$ (12)

Hence, we can obtain:

$$C_{4}=\frac{1}{2}k_{e}\mu_{\text{ind}}r^{2}q$$ (13)

$$\mu_{\text{ind}}=\frac{2C_{4}}{k_{e}qr^{2}}$$ (14)

*k*_e_ is a constant of 8.988 × 10^9^ J m/C^2^ when $\varepsilon_{r}=1$, so we can calculate $\mu_{\text{ind}}$ based on *C*_4_, *q* and *r* for each of the metal ions investigated in the present study. The results are shown in Table 5. These values showed excellent agreement with the values in Table 3, validating the computational strategy employed in the present study.

**TABLE 5:** Calculated $\mu_{\text{ind}}$ and $\mu_{\text{tot}}$ for the first solvation shell water molecules of different metal ions based on Eq. 14.

	*C*_4_ (kcal/mol⋅Å^4^)	*r* (Å)*^a^*	$\mu_{\text{ind}}$ (D)	$\mu_{\text{tot}}$ (D)^b^
Mg^2+^	117	2.09	0.39	2.82
Zn^2+^	199	2.09	0.66	3.09
Al^3+^	363	1.88	0.99	3.42
Fe^3+^	429	2.03	1.00	3.43
In^3+^	330	2.16	0.68	3.11

a Referred to the IOD values in Table 1.

b By summing $\mu_{\text{per}}$ of the OPC3 water model (2.43 D) and $\mu_{\text{ind}}$.

### Implications to Metalloproteins

Previously, we found that the 12-6 model could not reproduce the experimental HFE and IOD values simultaneously when metal ion has a charge of +2 or higher. We attributed this deficiency to the overlook of the ion-induced dipole interaction and developed the 12-6-4 model accordingly. Encouragingly, the 12-6-4 model could simultaneously reproduce the experimental HFE and IOD values for various metal ions (Li et al., 2013; Li and Merz, 2014; Li et al., 2015a; Li et al., 2015b). Moreover, we also found that the 12-6-4 model could provide increased stability than the 12-6 model when simulating a metalloprotein system (Li et al., 2021). In addition, Sengupta et al. have shown that the regular 12-6 model could not well simulate the chelate effect between ethylenediamine and metal ions, while the 12-6-4 model was able to well model this effect after parameter optimization (Sengupta et al., 2018). Furthermore, Song et al. showed that the 12-6-4 model could well simulate metal ion binding to a protein active site after parameter refinement (Song et al., 2020). These results indicated that the 12-6-4 model can overcome the deficiency of the 12-6 model and provide an improved representation of the metal ion containing systems.

Recently, Macchiagodena et al. developed a 12-6 model for four-coordinated zinc sites in metalloproteins (Macchiagodena et al., 2019). In this model, they refitted the atomic charges of the coordinating groups in the ligating residues. In order to reproduce the mean distances between Zn^2+^ and the ligating atoms, they adjusted their Lennard-Jones parameters. In their final parameters, the charges of the ligating sulfur atoms increased by 6–19% when comparing to the charge of the corresponding sulfur atom in the AMBER force field, while the charges of the ligating nitrogen atoms increased by 75–134% when comparing to charges of the corresponding nitrogen atoms in the AMBER force field. Their parameters successfully reproduced the experimentally determined mean distances between Zn^2+^ and the ligating atoms and provided Zn^2+^ binding affinities which correlated well with the experimentally determined dissociation constants. The strategy employed in their study is similar to that was used in the present work which considers the polarization effect by adjusting the atomic charges of the coordinating groups. And this strategy could improve the representation of the classical force field just as the 12-6-4 model did.

## Conclusion

Our previous research found that the widely used 12-6 Lennard-Jones nonbonded model for ions could not reproduce the experimental HFE and IOD values simultaneously when ion has a charge of +2 or higher (Li et al., 2013; Li et al., 2015a; Li et al., 2015b). We discussed that this deficiency arises from the overlook of the ion-induced dipole interaction, which is proportional to 1/*r* ^4^ where *r* is the atomic distance (Li and Merz, 2014). Through an $-C_{4}/r^{4}$ term which takes the ion-induced dipole interaction into account, we proposed and parameterized the 12-6-4 model for various ions (Li and Merz, 2014; Li et al., 2015a; Li et al., 2015b). Encouragingly, the 12-6-4 model could well reproduce the experimental HFE and IOD values at the same time (Li and Merz, 2014; Li et al., 2015a; Li et al., 2015b). However, we discovered a CN overestimation issue for some highly charged ions, and we discussed that this artifact arises from the fact that the current 12-6-4 model did not consider the increased repulsion among the first solvation water molecules of the metal ion (Li et al., 2015a; Li et al., 2021).

In the present study, we simulated multiple divalent and trivalent ions (Mg^2+^, Zn^2+^, Al^3+^, Fe^3+^, and In^3+^) by using the 12-6 model with adjusting the atomic charges in the first solvation shell water molecules to consider the ion-induced dipole effect. We showed that this strategy could well reproduce the experimental HFE and IOD values simultaneously for these ions and solve the CN overestimation issue of the 12-6-4 model for Fe^3+^ and In^3+^. In addition, we found that the enlarged magnitudes of the dipole moments of the first solvation shell water molecules in our simulations agreed well with previous AIMD simulations, further validating the current strategy. Besides, we derived the relationship between the *C*_4_ parameter and the induced dipole moment according to the physical principles. Moreover, the induced dipole moments calculated based on the *C*_4_ parameters showed good agreement with our simulation results. Finally, we discussed that both the 12-6-4 scheme and the 12-6 scheme with adjusting atomic charges could simulate the polarization effect in metalloprotein systems. Due to the fluctuating charge model also uses the 12-6 scheme with adjusting the atomic charges, we believe the present study can serve as a bridge between the 12-6-4 model and the fluctuating charge model.

## Data Availability

The original contributions presented in the study are included in the article/supplementary material, further inquiries can be directed to the corresponding author.

## References

[B1] Adamo C. Barone V. (1999). Toward Reliable Density Functional Methods without Adjustable Parameters: The PBE0 Model. J. Chem. Phys. 110, 6158–6170. 10.1063/1.478522

[B2] Andreini C. Bertini I. Rosato A. (2004). A Hint to Search for Metalloproteins in Gene banks. Bioinformatics 20, 1373–1380. 10.1093/bioinformatics/bth095 14962940

[B3] Becke A. D. (1988). Density-functional Exchange-Energy Approximation with Correct Asymptotic Behavior. Phys. Rev. A. 38, 3098–3100. 10.1103/physreva.38.3098 9900728

[B4] Becke A. D. (1993). Density‐functional Thermochemistry. III. The Role of Exact Exchange. J. Chem. Phys. 98, 5648–5652. 10.1063/1.464913

[B5] Bogatko S. A. Bylaska E. J. Weare J. H. (2010). First Principles Simulation of the Bonding, Vibrational, and Electronic Properties of the Hydration Shells of the High-Spin Fe3+ Ion in Aqueous Solutions. J. Phys. Chem. A. 114, 2189–2200. 10.1021/jp904967n 20078102

[B6] Cauët E. Bogatko S. Weare J. H. Fulton J. L. Schenter G. K. Bylaska E. J. (2010). Structure and Dynamics of the Hydration Shells of the Zn2+ Ion Fromab Initiomolecular Dynamics and Combinedab Initioand Classical Molecular Dynamics Simulations. J. Chem. Phys. 132, 194502. 10.1063/1.3421542 20499974

[B7] Cornell W. D. Cieplak P. Bayly C. I. Gould I. R. Merz K. M. Jr Ferguson D. M. (1995). A Second Generation Force Field for the Simulation of Proteins, Nucleic Acids, and Organic Molecules. J. Am. Chem. Soc. 117, 5179–5197. 10.1021/ja00124a002

[B8] Case D. A. Ben-Shalom I. Y. Brozell S. R. Cerutti D. S. Cheatham T. E. Cruzeiro V. W. D. (2020). Scan Francisco. AMBER 2020, University of California

[B9] Darden T. York D. Pedersen L. (1993). Particle Mesh Ewald: AnN⋅Log(N) Method for Ewald Sums in Large Systems. J. Chem. Phys. 98, 10089–10092. 10.1063/1.464397

[B10] De Vivo M. Masetti M. Bottegoni G. Cavalli A. (2016). Role of Molecular Dynamics and Related Methods in Drug Discovery. J. Med. Chem. 59, 4035–4061. 10.1021/acs.jmedchem.5b01684 26807648

[B11] Duan Y. Kollman P. A. (1998). Pathways to a Protein Folding Intermediate Observed in a 1-microsecond Simulation in Aqueous Solution. Science 282, 740–744. 10.1126/science.282.5389.740 9784131

[B12] Duarte F. Bauer P. Barrozo A. Amrein B. A. Purg M. Åqvist J. (2014). Force Field Independent Metal Parameters Using a Nonbonded Dummy Model. J. Phys. Chem. B 118, 4351–4362. 10.1021/jp501737x 24670003 PMC4180081

[B13] Dyke T. R. Muenter J. S. (1973). Electric Dipole Moments of Low J States of H2O and D2O. J. Chem. Phys. 59, 3125–3127. 10.1063/1.1680453

[B14] Eisenberg D. S. Kauzmann W. (1969). The Structure and Properties of Water. New York: Oxford University Press.

[B15] Essmann U. Perera L. Berkowitz M. L. Darden T. Lee H. Pedersen L. G. (1995). A Smooth Particle Mesh Ewald Method. J. Chem. Phys. 103, 8577–8593. 10.1063/1.470117

[B16] Hariharan P. C. Pople J. A. (1973). The Influence of Polarization Functions on Molecular Orbital Hydrogenation Energies. Theoret. Chim. Acta 28, 213–222. 10.1007/bf00533485

[B17] Hehre W. J. Ditchfield R. Pople J. A. (1972). Self-consistent Molecular Orbital Methods. XII. Further Extensions of Gaussian-type Basis Sets for Use in Molecular Orbital Studies of Organic Molecules. J. Chem. Phys. 56, 2257–2261. 10.1063/1.1677527

[B18] Hollingsworth S. A. Dror R. O. (2018). Molecular Dynamics Simulation for All. Neuron 99, 1129–1143. 10.1016/j.neuron.2018.08.011 30236283 PMC6209097

[B19] Izadi S. Onufriev A. V. (2016). Accuracy Limit of Rigid 3-point Water Models. J. Chem. Phys. 145, 074501. 10.1063/1.4960175 PMC4991989 27544113

[B20] Kepp K. P. (2012). Bioinorganic Chemistry of Alzheimer's Disease. Chem. Rev. 112, 5193–5239. 10.1021/cr300009x 22793492

[B21] Kollman P. (1993). Free Energy Calculations: Applications to Chemical and Biochemical Phenomena. Chem. Rev. 93, 2395–2417. 10.1021/cr00023a004

[B22] Lee C. Yang W. Parr R. G. (1988). Development of the Colle-Salvetti Correlation-Energy Formula into a Functional of the Electron Density. Phys. Rev. B 37, 785–789. 10.1103/physrevb.37.785 9944570

[B23] Lee T.-S. Allen B. K. Giese T. J. Guo Z. Li P. Lin C. (2020). Alchemical Binding Free Energy Calculations in AMBER20: Advances and Best Practices for Drug Discovery. J. Chem. Inf. Model. 60, 5595–5623. 10.1021/acs.jcim.0c00613 32936637 PMC7686026

[B24] Li P. Merz K. M. Jr. (2014). Taking into Account the Ion-Induced Dipole Interaction in the Nonbonded Model of Ions. J. Chem. Theor. Comput. 10, 289–297. 10.1021/ct400751u PMC3960013 24659926

[B25] Li P. Merz K. M. (2017). Metal Ion Modeling Using Classical Mechanics. Chem. Rev. 117, 1564–1686. 10.1021/acs.chemrev.6b00440 28045509 PMC5312828

[B26] Li P. Roberts B. P. Chakravorty D. K. Merz K. M. Jr. (2013). Rational Design of Particle Mesh Ewald Compatible Lennard-Jones Parameters for +2 Metal Cations in Explicit Solvent. J. Chem. Theor. Comput. 9, 2733–2748. 10.1021/ct400146w PMC3728907 23914143

[B27] Li P. Song L. F. Merz K. M. Jr. (2015a). Parameterization of Highly Charged Metal Ions Using the 12-6-4 LJ-type Nonbonded Model in Explicit Water. J. Phys. Chem. B 119, 883–895. 10.1021/jp505875v 25145273 PMC4306492

[B28] Li P. Song L. F. Merz K. M. Jr. (2015b). Systematic Parameterization of Monovalent Ions Employing the Nonbonded Model. J. Chem. Theor. Comput. 11, 1645–1657. 10.1021/ct500918t 26574374

[B29] Li Z. Song L. F. Li P. Merz K. M. Jr. (2021). Parametrization of Trivalent and Tetravalent Metal Ions for the OPC3, OPC, TIP3P-FB, and TIP4P-FB Water Models. J. Chem. Theor. Comput. 17, 2342–2354. 10.1021/acs.jctc.0c01320 PMC8173366 33793233

[B30] Li Z. Song L. F. Li P. Merz K. M. Jr. (2020). Systematic Parametrization of Divalent Metal Ions for the OPC3, OPC, TIP3P-FB, and TIP4P-FB Water Models. J. Chem. Theor. Comput. 16, 4429–4442. 10.1021/acs.jctc.0c00194 PMC8173364 32510956

[B31] Lightstone F. C. Schwegler E. Hood R. Q. Gygi F. Galli G. (2001). A First Principles Molecular Dynamics Simulation of the Hydrated Magnesium Ion. Chem. Phys. Lett. 343, 549–555. 10.1016/s0009-2614(01)00735-7

[B32] Lindorff-Larsen K. Piana S. Dror R. O. Shaw D. E. (2011). How Fast-Folding Proteins Fold. Science 334, 517–520. 10.1126/science.1208351 22034434

[B33] Macchiagodena M. Pagliai M. Andreini C. Rosato A. Procacci P. (2019). Upgrading and Validation of the AMBER Force Field for Histidine and Cysteine Zinc(II)-Binding Residues in Sites with Four Protein Ligands. J. Chem. Inf. Model. 59, 3803–3816. 10.1021/acs.jcim.9b00407 31385702

[B34] Marcus Y. (1988). Ionic Radii in Aqueous Solutions. Chem. Rev. 88, 1475–1498. 10.1021/cr00090a003

[B35] Marcus Y. (1991). Thermodynamics of Solvation of Ions. Part 5.-Gibbs Free Energy of Hydration at 298.15 K. J. Chem. Soc. Faraday Trans. 87, 2995–2999. 10.1039/ft9918702995

[B36] Miyamoto S. Kollman P. A. (1992). SETTLE: an Analytical Version of the SHAKE and RATTLE Algorithm for Rigid Water Models. J. Comput. Chem. 13, 952–962. 10.1002/jcc.540130805

[B37] Frisch M. J. Schlegel G. W. T., H. B. Scuseria G. E. Robb M. A. Cheeseman J. R. Scalmani G. (2019). Gaussian 16, Revision C. 01. Gaussian, Inc., Wallingford CT).

[B38] Perdew J. P. Burke K. Ernzerhof M. (1996). Generalized Gradient Approximation Made Simple. Phys. Rev. Lett. 77, 3865–3868. 10.1103/physrevlett.77.3865 10062328

[B39] Perdew J. P. Burke K. Ernzerhof M. (1997). Generalized Gradient Approximation Made Simple [Phys. Rev. Lett. 77, 3865 (1996)]. Phys. Rev. Lett. 78, 1396. 10.1103/physrevlett.78.1396 10062328

[B40] Peters M. B. Yang Y. Wang B. Füsti-Molnár L. Weaver M. N. Merz K. M. Jr. (2010). Structural Survey of Zinc-Containing Proteins and Development of the Zinc AMBER Force Field (ZAFF). J. Chem. Theor. Comput. 6, 2935–2947. 10.1021/ct1002626 PMC2941202 20856692

[B41] Sakharov D. V. Lim C. (2009). Force fields Including Charge Transfer and Local Polarization Effects: Application to Proteins Containing Multi/heavy Metal Ions. J. Comput. Chem. 30, 191–202. 10.1002/jcc.21048 18566982

[B42] Salomon-Ferrer R. Götz A. W. Poole D. Le Grand S. Walker R. C. (2013). Routine Microsecond Molecular Dynamics Simulations with AMBER on GPUs. 2. Explicit Solvent Particle Mesh Ewald. J. Chem. Theor. Comput. 9, 3878–3888. 10.1021/ct400314y 26592383

[B43] Sengupta A. Li Z. Song L. F. Li P. Merz K. M. Jr. (2021). Parameterization of Monovalent Ions for the OPC3, OPC, TIP3P-FB, and TIP4P-FB Water Models. J. Chem. Inf. Model. 61, 869–880. 10.1021/acs.jcim.0c01390 33538599 PMC8173365

[B44] Sengupta A. Seitz A. Merz K. M. (2018). Simulating the Chelate Effect. J. Am. Chem. Soc. 140, 15166–15169. 10.1021/jacs.8b09371 30381949

[B45] Song L. F. Sengupta A. Merz K. M. Jr. (2020). Thermodynamics of Transition Metal Ion Binding to Proteins. J. Am. Chem. Soc. 142, 6365–6374. 10.1021/jacs.0c01329 32141296

[B46] Stephens P. J. Devlin F. J. Chabalowski C. F. Frisch M. J. (1994). Ab Initio calculation of Vibrational Absorption and Circular Dichroism Spectra Using Density Functional Force fields. J. Phys. Chem. 98, 11623–11627. 10.1021/j100096a001

[B47] Thomson A. J. Gray H. B. (1998). Bio-inorganic Chemistry. Curr. Opin. Chem. Biol. 2, 155–158. 10.1016/s1367-5931(98)80056-2 9667942

[B48] Towns J. Cockerill T. Dahan M. Foster I. Gaither K. Grimshaw A. (2014). XSEDE: Accelerating Scientific Discovery. Comput. Sci. Eng. 16, 62–74. 10.1109/mcse.2014.80

[B49] Waldron K. J. Robinson N. J. (2009). How Do Bacterial Cells Ensure that Metalloproteins Get the Correct Metal?. Nat. Rev. Microbiol. 7, 25–35. 10.1038/nrmicro2057 19079350

[B50] Woodson S. A. (2005). Metal Ions and RNA Folding: a Highly Charged Topic with a Dynamic Future. Curr. Opin. Chem. Biol. 9, 104–109. 10.1016/j.cbpa.2005.02.004 15811793

[B51] Zhang J. Yang W. Piquemal J.-P. Ren P. (2012). Modeling Structural Coordination and Ligand Binding in Zinc Proteins with a Polarizable Potential. J. Chem. Theor. Comput. 8, 1314–1324. 10.1021/ct200812y PMC3383645 22754403

[B52] Zwanzig R. W. (1954). High‐Temperature Equation of State by a Perturbation Method. I. Nonpolar Gases. J. Chem. Phys. 22, 1420–1426. 10.1063/1.1740409

